# “Publish or perish”—presentations at annual national orthopaedic meetings and their correlation with subsequent publication

**DOI:** 10.1186/s13018-015-0203-y

**Published:** 2015-05-07

**Authors:** Zubin J Daruwalla, Sumon S Huq, Keng L Wong, Pei Y Nee, Diarmuid P Murphy

**Affiliations:** Department of Orthopaedic Surgery, National University Hospital, 5 Lower Kent Ridge Road, Singapore, 119074 Singapore; Yong Loo Lin School of Medicine, National University Singapore, Singapore, 117597 Singapore

## Abstract

**Background:**

Presentation of research at annual national orthopaedic conferences not only serves as a forum for the dissemination of knowledge but is also often a requirement of orthopaedic training programmes. The expected outcome is publication in a peer-reviewed journal. However, publication rates vary for a variety of reasons. The objective of this study was to determine publication rates of presentations from our local Singapore Orthopaedic Association (SOA) annual scientific meeting (ASM) and some of the potential associated factors. We also compared our findings to equivalent meetings worldwide to assess value of scientific content of various orthopaedic conferences.

**Methods:**

All presentations of six SOA ASMs were entered into a database. Using presentation titles, author names and keywords in PubMed and Google Scholar, we determined how many presentations progressed to publication in a peer-reviewed journal. Various comparisons were made to determine factors that could influence publication rates. A comparison with national orthopaedic meetings of America, United Kingdom, Ireland, Australia, Germany, Turkey and Brazil was also conducted.

**Results:**

Excluding the ASMs with less than 4 years of follow-up, the publication rate was 35.8%. Both podium and international presenters were found to have significantly higher publication rates than poster and local presenters, respectively, while basic science and clinical research were found to have equivalent rates. Publication rates from other countries’ national conferences ranged between 26.6% and 58.1%.

**Conclusions:**

We suggest that the quality of a presentation is related to its subsequent publication in a peer-reviewed journal. Our findings support the general consensus that the annual meeting of the American Academy of Orthopaedic Surgeons (AAOS) is the gold standard for the dissemination of orthopaedic knowledge updates and advancements in our specialty. Each national orthopaedic association could determine the ratio of “presentations at ASM” to “publication within five years of presentation” and use this as a measure of their annual conference’s impact on the addition and advancement to the orthopaedic literature. This tool may in turn assist clinicians in determining which meetings to attend.

## Introduction

Presentation of research at annual national and international orthopaedic conferences not only serves as a forum for the dissemination of knowledge [1] but is also often a requirement of orthopaedic training programmes. In Singapore, it is a prerequisite of the Residency Advisory Committee (RAC) that all orthopaedic residents must have at least two podium presentations at the Singapore Orthopaedic Association (SOA) annual scientific meeting (ASM) during their 6 years of training. It is expected that these presentations of basic science and clinical research will form the basis of a subsequent publication in a peer-reviewed journal. Indeed, the phrase “publish or perish” is still very much to the fore with residents often expected to have a specified number of publications in a peer-reviewed journal prior to exiting. This is seen as the gold standard for disseminating scientific research as published papers will have gone through more stringent peer-reviewed processes than conference presentations. In fact, it is recommended that papers considered for publication should not cite conference abstracts for this reason [2]. Despite this, however, even major orthopaedic textbooks may reference conference abstracts for more than half their content [3]. This makes it important to ensure that the information shared at scientific meetings is as accurate as possible. Although publication rates vary from one conference to another for a variety of reasons, measuring the proportion of abstracts that go on to full publication may be a good way to measure the scientific value of a given conference. The objective of our study was to determine the publication rate for our national orthopaedic meeting, the SOA ASM, and investigate potential predictive factors for subsequent publication. We also aimed to make a comparison to similar meetings worldwide [1,3-11] as a rough marker of the quality of research on display.

## Materials and methods

All podium and poster presentations from the SOA ASM of six annual conferences, between 2007 and 2013 with the exception of 2008, were obtained. Abstract titles and author names were then entered into a database. For each abstract, the year of presentation was noted along with whether the work was presented as a podium or poster presentation and whether the work presented was from a local or international source. Orthopaedic subspecialties which were relevant to the abstract were also recorded. Author names were then typed into PubMed in order to identify potential corresponding publications. Abstract keywords were combined with author names, using the Boolean term “AND”, in order to narrow our search down if an author had more than one publication. The process was repeated for all possible keywords until either a match was found or until all combinations were exhausted. The process was then repeated using Google Scholar if a publication was not found. From the available data, *Χ*^2^ testing was then used to explore whether publication rates were influenced by the mode of abstract presentation (podium vs. poster), the source of the research (local vs. international) and the type of research conducted (basic science vs. clinical research). Subspecialties were also explored as potential factors influencing likeliness of publication. Factors that were seen as potential predictors of publication were then entered together into a multivariate, binary logistic regression model to further confirm their predictive value with corrections made for potential confounding effects.

For each published abstract, the month and year of publication were recorded. From this, the time from conference presentation to publication was calculated in months. For articles that were published prior to their respective conference presentations, the time from publication to presentation was calculated in a similar way with the number of months given a negative value and included in further analysis. Some aspects of the data analysis would also discard abstracts that were published before a subsequent conference presentation. The journal in which each full paper was published was also recorded as well as its impact factor (IF) for the year in which the respective article was published. Data on the IF was first sourced from Journal Citation Reports® (JCR) [12] and then from the SCImago Journal and Country Rank (SJR) website [13] if the relevant data could not be obtained from the former. If no IF value could be found for a particular journal, it was given a value of zero and included in subsequent analysis. Finally, using binary logistic regression, a comparison of publication rates was made with other countries’ national orthopaedic scientific meetings around the world [1,3-11] in which the relevant data was published in the current literature to compare the value of attending various orthopaedic meetings. For those meetings that had more than one publication relevant to this study, the most recent publication was used.

All statistical analysis was performed using IBM® SPSS® Statistic Version 21.0 (IBM Corp. 2012) which included descriptive as well as inferential statistics. Normal distribution of data was inspected visually with the use of histograms and statistically from *z* scores calculated by dividing the measured skewness value of a data set by its standard error, *z* scores between −1.96 and 1.96 indicating normally distributed data [14]. Comparison of two or more normally distributed data sets was used using Student’s *t*-test and one-way ANOVA, respectively. Mann–Whitney *U* testing was used to compare data sets that were non-normally distributed. Analysis of dichotomous data was done using *Χ*^2^ tests with odds ratios (OR) calculated along with 95% confidence intervals (CI). Fisher’s exact test was used as an alternative when expected counts were seen to be less than five. Differences were seen as significant when *p* < 0.05.

## Results

**Figure 1:**
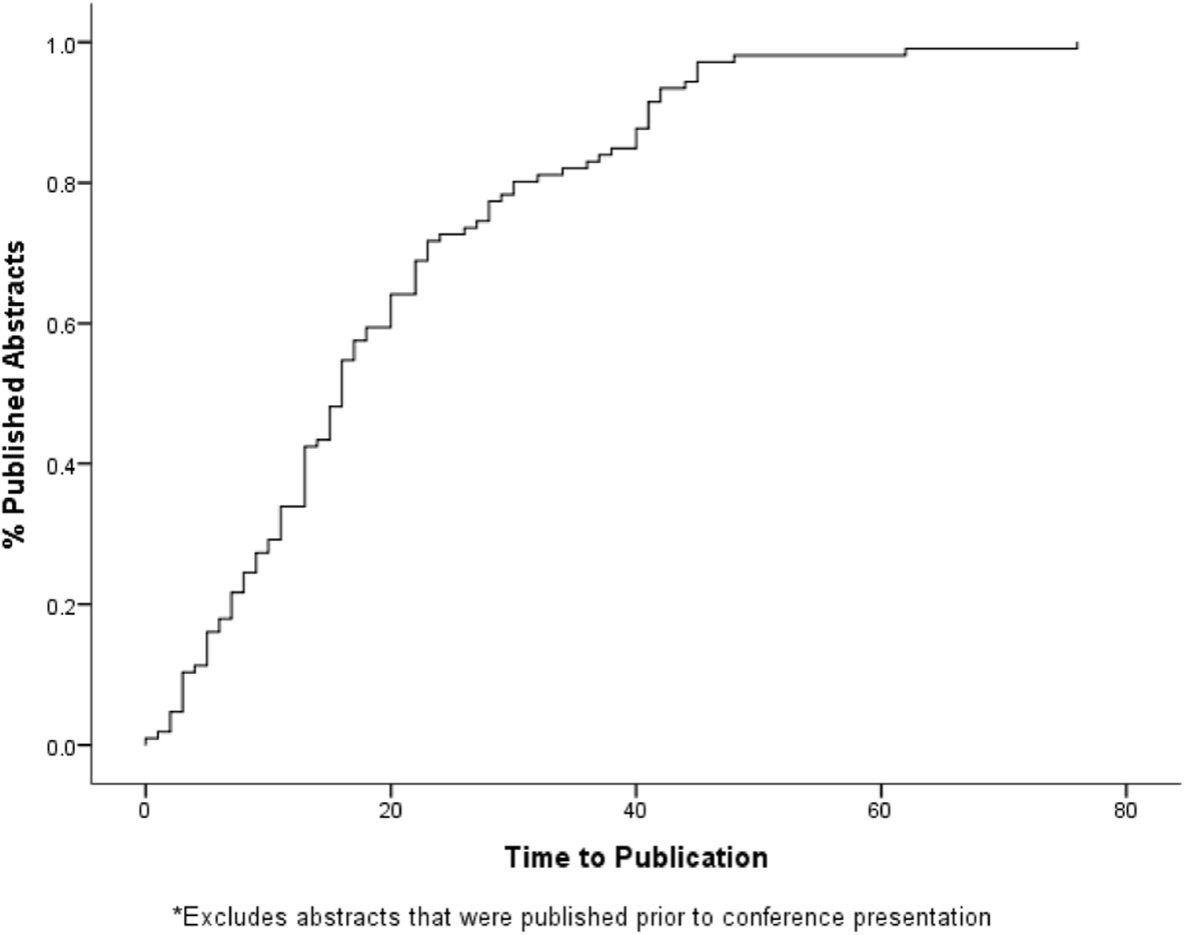
Survivorship graph illustrating distribution of time taken for publication.

**Figure 2:**
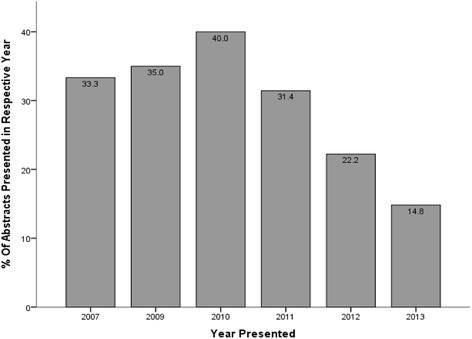
Publication rates from the respective SOA conferences.

A total of 443 abstracts were presented in all the conferences studied, of which 125 (28.2%) were published, 20 (16.0%) prior to presentation with 103 (98.1%) of the remaining 105 within 4 years of presentation (average 16.5 months) as can be seen in Figure 1. The majority of published studies were either retrospective (44.8%) or prospective (29.6%) as can be seen in Table 1. Figure 2 illustrates the publication rates for each annual conference included in our study. This shows a drop-off for the 2011, 2012 and 2013 conferences which, when combined, were shown to have a significantly lower publication rate than the rest of the conferences combined (35.8% vs. 22.4%; OR 1.928; CI 1.269–2.929; *p* = 0.02).

**Table 1:** **Numbers of various study types within published abstracts**

	**Numbers**	**Percent**
Retrospective case series	56	44.8
Prospective case series	37	29.6
Case report	10	8.0
Randomized controlled trial	9	7.2
Cadaver	3	2.4
Cross-sectional	3	2.4
In vitro	2	1.6
Biomechanics	1	0.8
Case–control	1	0.8
Imaging	1	0.8
Prospective cohort	1	0.8
Systematic review	1	0.8
Total	125	100.0

Tables 2 and 3 summarize the analysis of potential predictive factors for publication. Initial analysis revealed only three potentially significant factors that may predict likelihood for publication. When these factors were put together into a multivariate regression model, they were still found to be significant. From this analysis, it was shown that podium presentations were more than twice as likely to be published compared to poster presentations (OR 2.288; CI 1.118–4.684; *p* = 0.02) and international presenters were more likely to have their work published than local presenters (OR 1.741; CI 1.067–2.842; *p* = 0.027). Analysis of the third significant predictive factor suggested that trauma papers were less likely to be published compared to other subspecialties (OR 0.530; CI 0.310–0.904; *p* = 0.02).

**Table 2:** **Analysis of subspecialties as potential predictive factors for publication**

**Specialty**	**No. published/no. presented (%)**	**% published abstracts** ^**a**^	**% unpublished abstracts** ^**b**^	**Odds ratio (OR)**	**Confidence interval (CI)**	***p*** **value**
Foot/ankle	18/56 (32.1)	14.4	11.9	1.205	0.716–2.029	0.485
Hand/wrist	4/6 (66.7)	3.2	0.6	5.088	0.944–27.429	0.056^c^
Hip/knee	54/209 (25.8)	43.2	48.7	0.886	0.704–1.116	0.293
Infectious disease	2/19 (10. 5)	1.6	5.3	0.299	0.070–1.277	0.080
Metabolic	3/15 (20.0)	2.4	3.8	0.636	0.174–2.261	0.472
Oncology	9/20 (45.0)	7.2	3. 5	2.081	0.884–4.901	0.088
Paediatrics	7/24 (29.2)	5.6	5.3	1.048	0.445–2.464	0.915
Shoulder/elbow	15/39 (38. 5)	12.0	7. 5	1. 590	0.863–2.929	0.137
Spine	13/51 (25.5)	10.4	11.9	0.870	0.480–1.578	0.646
Sports/arthroscopy	9/38 (23.7)	7.2	9.1	0.790	0.385–1.620	0. 516
Trauma	21/111 (18.9)	16.8	28.3	0. 530^d^	0.310–0.904^d^	0.02^d^
Others	2/8 (25.0)	1.6	1.9	0.848	0.173–4.145	1.000^c^

^a^Out of all 125 published abstracts, ^b^out of 318 unpublished abstracts, ^c^Fisher’s exact test used, ^d^after multivariate binary logistic regression analysis.

**Table 3:** **Analysis of other potential predictive factors for publication**

**Factor**	**% published**	**OR**	**CI**	***p*** **value**
Podium vs. poster				
Podium	30.3	2.288^a^	1.118–4.684^a^	0.020^a^
Poster	15.6			
International vs. local				
International	39.1	1.741^a^	1.067–2.842^a^	0.027^a^
Local	25.4			
Clinical vs. basic science				
Basic science	32.1	1.221	0.537–2.777	0.633
Clinical	28.0			

^a^After multivariate binary logistic regression analysis.

**Figure 3:**
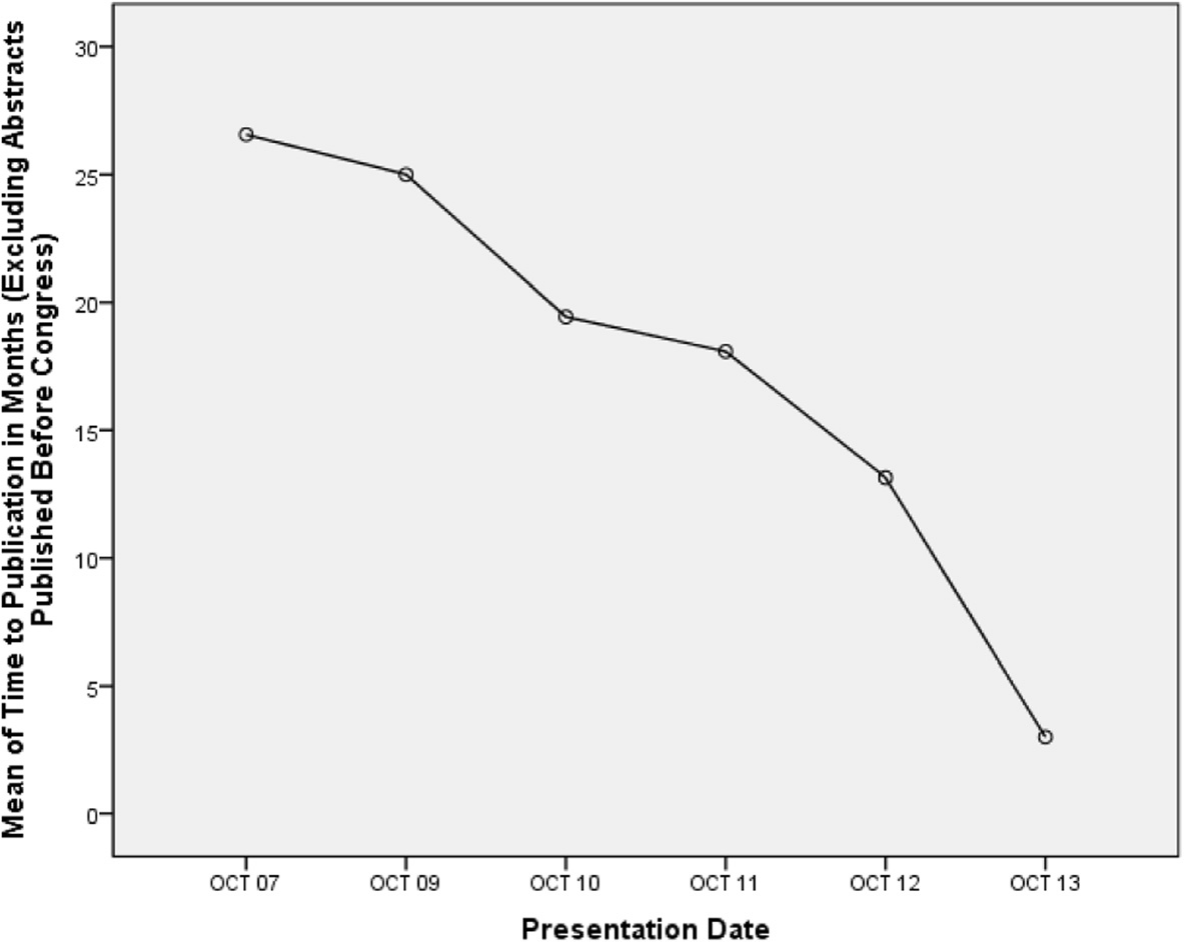
Graph illustrating mean time to publication for each year investigated, showing a decrease in time to publication in more recent years.

Distribution of time to publication revealed the data to be skewed for the whole group, although they were normally distributed for individual conferences. Overall median time, in months, to publication for all published papers was 13 (range −73 to 76). Subsequent analysis excluded papers that were published prior to conference presentation which increased the median to 16.5 (range 0–76). One-way ANOVA analysis revealed significant differences in the time to publication between different years of the conference (*p* < 0.001) with rates falling in more recent conferences as shown in Figure 3. Further analysis seemed to show that abstracts that were basic science or clinical research papers, podium or poster presentations or from local or international presenters had no influence over the time to publication (*p* = 0.137, 0.241 and 0.46, respectively).

Published abstracts were disseminated in 54 peer-reviewed journals overall with the top three being the Journal of Orthopaedic Surgery (Hong Kong) (14.0%), the Singapore Medical Journal (10.4%) and the British Journal of Bone and Joint Surgery (7.2%) as shown in Table 4. Of 125 published abstracts, 112 (89.6%) were found in journals that had IF information which left 13 (10.4%) with no IF information according to the methods used in this study. Of the articles in journals with an IF, 82 (73.2%) had the relevant information found in JCR [12] with 30 (26.8%) having the relevant data in SJR [13]. Table 5 summarizes these findings. The median impact factor achieved for all published articles was 0.96 (0–25.12) with no differences seen in the annual averages (*p* = 0.291). Further analysis also showed that abstracts that were basic science or clinical research papers, podium or poster presentations or from local or international presenters had no predictive value in determining the IF of a published paper (*p* = 0.271, 0.957 and 0.598, respectively).

**Table 4:** **Journals in which published abstracts were disseminated**

**Journal**	**Frequency**	**Percent**
J Orthop Surg (Hong Kong)	14	11.2
Singapore Med J	13	10.4
J Bone Joint Surg Br	9	7.2
J Arthroplasty	7	5.6
Ann Acad Med Singapore	6	4.8
Knee Surg Sports Traumatol Arthrosc	6	4.8
J Orthop Surg Res	4	3.2
Eur Spine J	3	2.4
Indian J Orthop	3	2.4
J Bone Joint Surg Am	3	2.4
Knee	3	2.4
Spine (Philadelphia, Pa. 1976)	3	2.4
Acta Orthop Belg	2	1.6
Arthroscopy	2	1.6
Clin Biomech (Bristol, Avon)	2	1.6
Foot Ankle Int	2	1.6
Foot Ankle Spec	2	1.6
J Knee Surg	2	1.6
J Orthop	2	1.6
Malaysian Orthop J	2	1.6
Proceedings of Singapore Healthcare	2	1.6
Am J Emerg Med	1	0.8
Arthritis	1	0.8
Clinical Anatomy	1	0.8
Colloids Surf B Biointerfaces	1	0.8
Curr Orthop Prac	1	0.8
Diabet Foot Ankle	1	0.8
Eur J Emerg Med	1	0.8
Eur J Orthop Surg Traumatol	1	0.8
Foot Ankle Surg	1	0.8
Hip Int	1	0.8
Int J Rheum Dis	1	0.8
Int J Surg Case Rep	1	0.8
Ir J Med Sci	1	0.8
J Biomech	1	0.8
J Biomed Opt	1	0.8
J Child Orthop	1	0.8
J Clin Rheumatol	1	0.8
J Foot Ankle Surg	1	0.8
J Med Assoc Thai	1	0.8
J Med Cases	1	0.8
J Paediatr Orthop	1	0.8
J Spinal Disord Tech	1	0.8
Jiangsu Medical Journal	1	0.8
Journal of Orthopedics, Trauma and Rehabilitation	1	0.8
Lancet Oncol	1	0.8
Med J Indones	1	0.8
OA Case Reports	1	0.8
Orthop Surg	1	0.8
Orthopedics	1	0.8
Osteoporos Int	1	0.8
Spine J	1	0.8
Surgeon	1	0.8
The Orthopedic Journal of China	1	0.8
Total	125	100.0

**Table 5:** **Impact factor source**

	**Frequency**	**% published abstracts**
Journal citation reports	82	65.6
Scientific journal rankings	30	24.0
No impact factor	13	10.4
Total	125	100.0

Comparisons were finally made with the publication rates of other countries’ national orthopaedic association meetings that had relevant published data. These were namely from the American Academy of Orthopaedic Surgeons (AAOS) [4,5], British Orthopaedic Association (BOA) [6], Irish Orthopaedic Association (IOA) [1], Australian Orthopaedic Association (AOA) [7], German Society of Orthopaedics and Trauma Surgery (GSOTS) [8], Turkish Orthopaedics and Traumatology Congress (TOTC) [9] and the Congresso Brasileiro de Ortopedia (CBOT) [10]. Table 6 summarizes the publications for all these respective meetings. Compared to other conferences included in this study, publication rates for the SOA meeting showed no significant differences to all except the AAOS [5] (OR 3.523; CI 2.737–4.534; *p* < 0.001), BOA [6] (OR 1.451; CI 1.004–2.096; *p* = 0.048) and GSOTS [8] (OR 1.367; CI 1.020–1.832; *p* = 0.037). During the analysis of this data, the high publication rate of the AAOS [5] at 58.1% was looked at in more detail. This analysis showed a significantly higher publication rate from the AAOS [5] than all the rest of the meetings included in this study (*p* values were all <0.001).

**Table 6:** **Comparison of publications rates with other national orthopaedic meetings**

**Conference (year)**	**Total abstracts**	**Abstracts published**	**% published**	**OR**	**CI**	***p*** **value**
SOA (2007, 2009–2013)	443	125	28.2	1.000	–	–
AAOS (2001)	756	439	58.1	3. 523	2.737–4.534	<0.001
BOA (2001)	179	65	36.3	1.451	1.004–2.096	0.048
IOA (2002–2005)	203	66	32. 5	1.226	0.856–1.755	0.267
AOA (1998)	200	62	31.0	1.143	0.794–1.645	0.472
GSOTS (2003)	392	137	34.9	1.367	1.020–1.832	0.037
TOTC (2007)	770	227	29. 5	1.064	0.821–1.377	0.641
CBOT (2007)	653	174	26.6	0.924	0.705–1.211	0. 567

*SOA* Singapore Orthopaedic Association, *AAOS* American Academy of Orthopaedic Surgeons, *BOA* British Orthopaedic Association, *IOA* Irish Orthopaedic Association, *AOA* Australian Orthopaedic Association, *GSOTS* German Society of Orthopaedics and Trauma Surgery, *TOTC* Turkish Orthopaedic and Traumatology Congress, *CBOT* Congresso Brasileiro de Ortopedia.

## Discussion

This study showed that, with a publication rate of 28.2% (or 35.8% for conferences with at least 4 years of follow-up), the majority of abstracts presented at the annual SOA scientific meeting will not progress to full publication. This is in keeping with most other major national orthopaedic association conferences, although present literature suggests that rates between meetings vary and range between 26.6% and 58.1% [1,3-11]. It should be noted, however, that the previously mentioned 58.1% was at 10 years post-presentation with the figure for the same AAOS meeting being 49% at 5 years [5]. Our local figure of 28.2% is thus likely an underestimate that can be attributed to the publication rate drop-off for the 2011, 2012 and 2013 conferences as this only gives a 3, 2 and 1-year post-presentation period, respectively, to check for publications when in fact we have shown that the majority of publications occur within 4 years and other studies quoting publication rates up to 10 years [5]. This can be clearly understood by noting the 40% publication rate from the 2010 SOA ASM at 4 years. Interestingly, however, a significantly shorter time to publication of presentations from these same three mentioned years compared to earlier years was noted and may be worth further investigating as this may reflect research of higher quality.

While we were unable to calculate statistical significance because of the unavailability of the necessary data, we did note that the majority of published studies were either retrospective or prospective. Podium presentations and international presenters were found to have a higher chance of being published than poster presentations and local presenters, respectively. Our findings regarding podium presentations being twice as likely to be published compared to poster presentations support data from other meetings which show twice [9] and thrice [10] the chance of the same. While trauma papers were found to be less likely published compared to other subspecialties, this is especially surprising considering that while general orthopaedic meeting publication rates vary as mentioned earlier, subspecialty conferences have been found to quote publication rates ranging between 40% and 64% [15-18], the highest being in trauma [20].

The median time to publication after presentation was found to be 16.5 months and ranged between 0 and 76 months. In contrast to other studies [5], we found that none of the potential factors we analysed, including type of study, source or form of presentation, influenced this. With regard to impact factor, the median achieved for all published articles was 0.96 and ranged between 0 and 25.12. Again, we found that none of the potential factors we analysed had any predictive value in determining the IF of a published paper but could not find anything else in the literature to support our observation.

At first glance, the average publication rate of our SOA ASMs of 28.2% may seem lacklustre. It must be remembered that this figure is likely an underestimate for reasons previously mentioned and evidenced by a 40% figure in 2010. Furthermore, in comparison to seven other worldwide conferences included in this study, publication rates for the SOA ASM showed no significant differences to all except the AAOS [5] and GSOTS [8]. Again, this may not be a true reflection and may be attributed to the drop-off also previously mentioned. Table 7 shows this to be taken into account by excluding publication rate data from the SOA meetings of 2011, 2012 and 2013. This shows the publication rate from the remaining meetings to be 35.8%, a figure that is comparable to most national meetings within this study [1,6-9], including the BOA [6] and GSOT [8] as well as IOA [1] meetings and significantly higher than the publication rate for CBOT [10], although it was still found to be significantly lower than the AAOS meeting [5]. When looked at in more detail, further analysis unsurprisingly showed a significantly higher publication rate from the AAOS than all the rest of the meetings included in this study, thus potentially representing that perhaps the annual meeting of the AAOS has sole superior publication rates.

**Table 7:** **Comparison of publications rates with other national orthopaedic meetings (excluding data from SOA ASMs from 2011 to 2013)**

**Conference (year)**	**Total abstracts**	**Abstracts published**	**% published**	**OR**	**CI**	***p*** **value**
SOA (2007, 2009 and 2010)	193	69	35.8	1.000	–	–
AAOS (2001)	756	439	58.1	2.489	1.793–3.454	<0.001
BOA (2001)	179	65	36.3	1.025	0.671–1. 565	0.910
IOA (2002–2005)	203	66	32. 5	0.866	0.571–1.312	0.497
AOA (1998)	200	62	31.0	0.807	0. 530–1.229	0.318
GSOTS (2003)	392	137	34.9	0.966	0.673–1.384	0.849
TOTC (2007)	770	227	29. 5	0.751	0. 539–1.048	0.092
CBOT (2007)	653	174	26.6	0.653	0.464–0.919	0. 014

*SOA* Singapore Orthopaedic Association, *AAOS* American Academy of Orthopaedic Surgeons, *BOA* British Orthopaedic Association, *IOA* Irish Orthopaedic Association, *AOA* Australian Orthopaedic Association, *GSOTS* German Society of Orthopaedics and Trauma Surgery, *TOTC* Turkish Orthopaedic and Traumatology Congress, *CBOT* Congresso Brasileiro de Ortopedia.

While a number of barriers to full-text publication following presentation of abstracts at annual orthopaedic meetings exists [19], there is no reason why all national orthopaedic associations should not strive to achieve having the majority of presentations at their annual conferences published in peer-reviewed journals as in the cases of other major orthopaedic [5] as well as subspecialty meetings [18,20-23]. In our local context, planning of research with a focus on a non-trauma-related prospective or retrospective case series and ensuring one’s submission is accepted as a podium presentation that maximizes the chances of conversion to a publication in a peer-reviewed journal.

Perhaps the publication to the 5-year post-presentation ratio is something each national orthopaedic association around the world should determine as a measure of their annual conference’s impact on the addition and advancement to the field of orthopaedic literature. This tool may in turn assist clinicians in determining which meetings to attend. Our findings support the general consensus amongst many orthopaedic surgeons that the annual meeting of the AAOS is the gold standard for the dissemination of orthopaedic knowledge updates and advancements in our specialty. This statement is easily backed by their superior publication rates of 49% at 5 and 58% at 10 years [5]. It should however be noted that, as many studies presented will not pass the scrutiny of peer-review, the information presented at the AAOS annual meeting (or any other meeting for that matter) should not be used as the sole guide to clinical practice [4]. Finally, with the recent introduction of the Accreditation Council for Graduate Medical Education-International (ACGME-I) and its residency programmes in Singapore, perhaps we should also adopt the stringent guidelines for acceptance of papers to the AAOS annual meeting when reviewing abstracts for the SOA ASM. This should improve the quality of the scientific work being presented at a local level as well as raise the standards of orthopaedic research being performed in Singapore. Lastly, working with the ACGME-I and aiming to have collaborative meetings with the AAOS in Singapore with the hope of learning from our American counterparts and striving to raise our standards of research to match their own is a goal well within reach and well worth pursuing.

## Conclusions

We suggest that the quality of a presentation is related to its subsequent publication in a peer-reviewed journal. Our findings support the general consensus that the annual meeting of the AAOS is the gold standard for the dissemination of orthopaedic knowledge updates and advancements in our specialty. Each national orthopaedic association could determine the ratio of “presentations at ASM” to “publication within five years of presentation” and use this as a measure of their annual conference’s impact on the addition and advancement to the orthopaedic literature. This tool may in turn assist clinicians in determining which meetings to attend.
